# Experience with a Hybrid Procedure Involving Laparoscopic Fundoplication with Percutaneous Endoscopic Gastrostomy in Chronically Ill Children

**DOI:** 10.3390/jcm10194291

**Published:** 2021-09-22

**Authors:** Sohyun Yoon, Soo-Hong Kim, Yeoun Joo Lee, Jae Hong Park, Yong Hoon Cho, Jae Hun Chung

**Affiliations:** 1 Department of Pediatrics, Pusan National University Children’s Hospital, Yangsan 50612, Korea; drawingyoon@naver.com (S.Y.); jhongpark@pusan.ac.kr (J.H.P.); 2 Department of Surgery, Pusan National University Children’s Hospital, Yangsan 50612, Korea; soohongnara@hanmail.net (S.-H.K.); choyh70@pusan.ac.kr (Y.H.C.); drjh1013@gmail.com (J.H.C.)

**Keywords:** gastrostomy, fundoplication, child, percutaneous endoscopic gastrostomy

## Abstract

Gastrostomy with concurrent laparoscopic Nissen fundoplication (LNF) is often performed as a laparoscopic gastrostomy (LG) by surgeons. Since 2014, we started performing percutaneous endoscopic gastrostomy (PEG) as gastrostomy with LNF. This study aims to compare the outcomes of LG and PEG with LNF. Patients were recruited into two groups: LNF with LG (historical control) or PEG. Demographic data, operation time, time to start feeding, time to full feeding, length of hospital stay (LOS), and complications were compared between the groups. Fourteen patients underwent LNF with LG and 49 underwent LNF with PEG. The median age and body weight of patients were 4.25 years and 14.15 kg in the LG group and 2.58 years and 10.60 kg in the PEG group, respectively. Operation times were significantly shorter in the PEG group (1.81 vs. 2.61 h). The times to start feeding and full feeding as well as LOS were shorter in the PEG group. Nevertheless, complications were similar in both groups. In conclusion, PEG with LNF was associated with significantly shorter operation times, times to start feeding and reach full feeding, and LOS. PEG is a suitable method for LNF in chronically ill children.

## 1. Introduction

Proper nutrition is very important for the growth and development of children. However, children with severe neurological impairment or other chronic diseases are more likely to have swallowing difficulties, gastroesophageal reflux (GER), or esophageal hernia [1]. They are also more likely to have gastrointestinal symptoms such as reflux, nausea, and vomiting [1]. Nutritional deficiencies are very common and reported in approximately 70% of children with neurological impairment [2] because many of them cannot fulfill their nutritional requirements via oral administration. Such feeding difficulties can easily lead to malnutrition and poor disease prognosis.

If oral feeding is impossible or insufficient to fulfill nutritional requirements, nasogastric feeding tubes are used for enteral feeding. However, long-term use of a nasogastric feeding tube can cause problems such as tube dislocation, aspiration, nasopharyngeal ulcers, and esophageal ulcers. Therefore, many guidelines recommend performing a gastrostomy if enteral nutrition is required for more than one month [3]. In addition, chronically malnourished patients with GER or esophageal hiatus hernia often fail to achieve proper nutrition due to their reflux symptoms. Therefore, gastrostomy is often performed with fundoplication when GER is present.

Gastrostomy techniques include conventional surgical gastrostomy, laparoscopic gastrostomy (LG), percutaneous endoscopic gastroscopy (PEG), and laparoscopic-assisted PEG [4,5]. Laparoscopic Nissen fundoplication (LNF) is currently the method of choice in children with medically intractable GER and has been performed in many centers.

We previously performed LNF and LG in pediatric patients who needed simultaneous fundoplication and gastrostomy. However, since 2014, we started performing PEG in the operating room simultaneously with LNF, and LNF with PEG instead of LG has shown positive results in many children. This hybrid approach of LNF with PEG has been reported several times in children with neurological disorders [6,7]. However, no studies have specifically compared the clinical outcomes of this method to those of LNF with LG.

This study compares the outcomes between patients who received LNF with LG and those who received LNF with PEG. The primary outcome of this study is to compare the perioperative conditions and parameters at the time of surgery between the two groups. The secondary outcome of this study is to compare the complications and prognoses between the two groups.

## 2. Materials and Methods

### 2.1. Subjects

Between March 2012 and November 2019, patients aged <18 years who underwent LNF and gastrostomy with either LG or PEG were recruited at Pusan National University Children’s Hospital. Patients who underwent fundoplication or gastrostomy alone were excluded. Clinical information, including age, sex, body weight, height, underlying diseases, and conditions, were reviewed from electronic medical records. Data related to surgery, such as operation duration, times to start feeding and reach full feeding, and complications, were also retrospectively reviewed.

This study was approved by the Institutional Review Board of Pusan National University Yangsan Hospital (05-2020-043). As this study was designed in a retrospective manner, the IRB approved the waiver of the individuals’ informed consent.

### 2.2. Operative Technique

All procedures were performed under general anesthesia, and prophylactic antibiotics were administered prior to the operation. LNF was conducted in the same manner, regardless of whether it was performed with LG or PEG. 

**Figure 1:**
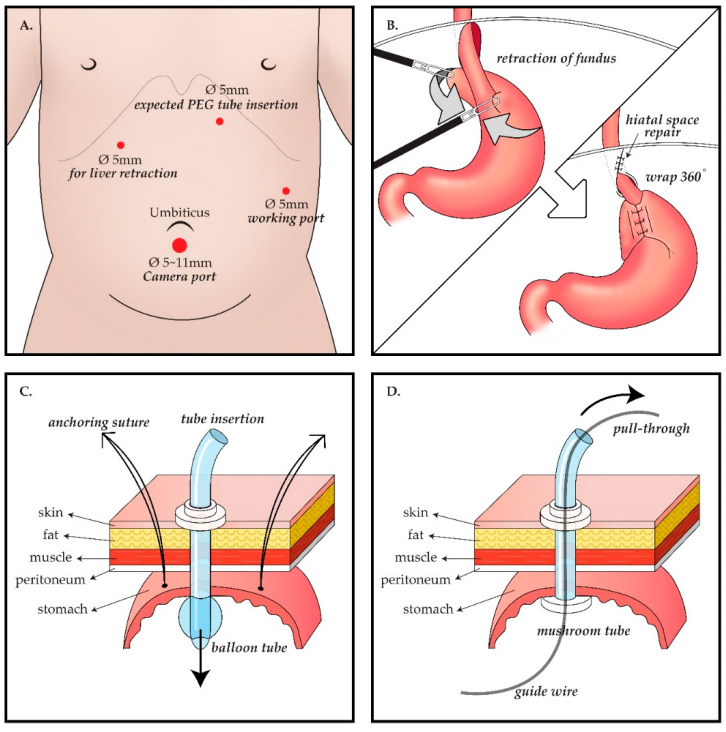
Schema of the operative procedure. Laparoscopic gastrostomy (LG) group follows A ⟶ B ⟶ C, whereas percutaneous endoscopic gastrostomy (PEG) group follows A ⟶ B ⟶ D. (**A**) Laparoscopic port placement: (i) 5 or 11 mm port inferior to the umbilicus for camera; (ii) 5 mm port to the lateral left of the umbilical line as a working port; (iii) 5mm port as another working port and this port would be the expected gastrostomy tube insertion site, at the left upper quadrant in the epigastrium, and (iv) 5 mm port at the right upper quadrant for retracting the liver. (**B**) Nissen fundoplication procedure: after mobilization of gastric fundus, the upper part of the gastric fundus is wrapped 360° around the lower end of the esophagus to tighten the lower esophageal sphincter. (**C**) LG procedure: Three anchoring sutures between the stomach and abdominal wall and a purse-string suture are applied around the gastrostomy site. Then, a high-profile ballooned gastrostomy tube is inserted. (**D**) PEG procedure: The stomach is punctured using a guide needle and a guide wire is inserted under guidance with both laparoscopy and endoscopy. By using a snare through the endoscope, the wire is captured and pulled out through the mouth. Following the wire from the mouth to the punctured stomach wall, a 20 Fr mushroom gastrostomy tube is introduced.

The location of laparoscopic port insertion and the LNF method differed depending on the extent of the patient’s scoliosis and hiatal hernia but were generally the same. Four ports were inserted 5 mm below the umbilicus (11 mm below the umbilicus if the patients weighed more than 30 kg), 5 mm to the lateral left of the umbilical line by the working port, by the location of the expected gastrostomy tube insertion site after laparoscopically confirming the site at the left upper quadrant in the epigastrium, and 5 mm into the right upper quadrant for retracting the liver. The LNF method is identical to the known Nissen fundoplication method. The upper part of the gastric fundus is wrapped 360° around the lower end of the esophagus to tighten the lower esophageal sphincter. If the hiatus is loose or a hiatal hernia is apparent, the surgeon separates the esophagus and hiatus, sutures the hiatus, and fixes the esophagus to the hiatus to prevent the esophagus from slipping to the chest, thereby securing most of its length. After peeling the short gastric arteries, the fundus around the gastroesophageal junction is wrapped and fixed with an interrupted suture using an Ethibond 4–0 (Figure 1). 

In the surgical gastrostomy group, after LNF, a small stab incision for gastrostomy was made for delivery out of the stomach through this site, and gastrostomy was performed. A high-profile ballooned gastrostomy tube (Mic, Kimberly-Clark) was inserted, and a purse-string suture with vicryl 4–0 was applied around the gastrostomy site. In the PEG group, after conventional LNF, endoscopy (GIF-XP260, Olympus) was performed, and PEG was performed using the pull-through technique. Initially, the tightness and status of the LNF were checked through the J turn of the endoscope in the stomach. A guide needle was inserted through the previous trocar insertion site. The adequacy of the gastrostomy location was assessed outside of the stomach by laparoscopy and inside the stomach by endoscopy. The stomach was then punctured using a guide needle. By using a snare through the endoscope, a thin wire guide needle was captured and pulled out through the mouth. Following the wire, a 20 Fr mushroom gastrostomy (Wilson-CookR, Wilson-Cook Medical Inc., Winston-Salem, NC, United States) tube was introduced. 

### 2.3. Study Methods

After dividing the patients into two groups (LNF with LG (historical control) and LNF with PEG), we compared demographic data, operation time, time to start feeding, time to full feeding, length of hospital stay (LOS), and complications. Complications were divided into minor complications, such as dislodged tubes, granuloma, local infection/inflammation, and leakage, and major complications, such as perforation, peritonitis, and malfunction. 

### 2.4. Statistical Analysis

Patient data are presented as the median. Between the two groups, continuous variables were compared using a Wilcoxon rank sum test, and categorical variables were compared using chi-squared or Fisher’s exact tests. A partial correlation was used to measure associations after adjusting for additional variables. 

Statistical analyses were performed using SPSS (25th version, SPSS Inc., Chicago, IL, USA). Statistical significance was set at *p* < 0.05.

## 3. Results

### 3.1. Patient Data

Sixty-three patients (33 male and 30 female) underwent LNF with simultaneous gastrostomy. A total of 14 patients (nine male and five female) underwent LG, and 49 patients (24 male and 25 female) underwent PEG (Table 1).

In the LG group, 14 patients (100%) had neurologic disorders and 10 patients (71%) had respiratory disorders. In the PEG group, 48 patients (98.0%) had neurological disor)s and 26 patients (53.1%) had respiratory disorders. Patients in both groups had various diseases, such as congenital heart diseases and metabolic diseases. Three (21.4%) and 11 (22.4%) patients underwent tracheostomy owing to chronic respiratory problems at the time of surgery in the LG and PEG groups, respectively (Table 2).

The median (range) ages and body weights of patients were 4.25 (0.68–18.26) years and 14.15 (3.9–25.7) kg and 2.58 (0.21–16.4) years and 10.60 (4.84–46.5) kg in the LG and PEG groups, respectively.

### 3.2. Immediate Postoperative Results

The median operation time was significantly shorter in the PEG group: 2.61 and 1.81 h in the LG and PEG groups, respectively (*p* = 0.001). After adjusting for the type of gastrostomy, operator, order of operation, age, body weight, and underlying diseases, no correlation with the operation time was observed. 

Two of the 14 patients in the LG group underwent chemoport implantation, and six of the 49 patients in the PEG group underwent other surgeries, including chemoport implantation, inguinal hernia repair operation, tracheostomy, ileostomy repair operation, adhesiolysis of previous abdominal surgery, pyloroplasty, or removal of a tracheal granuloma. In terms of surgery, hiatal hernias requiring repair were found in two (14.3%) and 22 (44.9%) cases in the LG and PEG groups, respectively.

The times to start feeding and reach full feeding (recommended volume) were shorter in the PEG group. The median (range) time to start feeding was 3.00 (1–18) days in the LG group and 2.00 (1–7) days in the PEG group (*p* = 0.0005). The median times to reach full feeding were 33.5 and 16.0 days in the LG and PEG groups, respectively (*p* = 0.043). The PEG group had a shorter median LOS (16.0 days) than the surgical group (33.5 days), but the difference was not statistically significant (*p* = 0.201) (Table 3).

### 3.3. Complications and Prognosis

Among the 63 patients, nine (14.3%) postoperative complications were reported. Local inflammation at the gastrostomy site was reported in one patient (7.1%) in the LG group and in seven patients (14.3%) in the PEG group. One case of peritonitis and one case of perforation occurred in the LG group (14.2%), while one case of peritonitis occurred in the PEG group (2.0%). One patient died of *Klebsiella pneumoniae* sepsis on the sixth postoperative day in the PEG group. All other patients with early complications recovered after supportive care. 

The mean follow-up duration was 48.9 ± 34.4 and 26.6 ± 16.5 months in the LG and PEG groups, respectively. During the long-term follow-up, one patient (7.1%) developed recurrent GER and required another LNF, while one patient (7.1%) died of complications from an underlying disorder in the LG group. In the PEG group, no patients experienced recurrent GER that required surgery, two patients (4.1%) underwent tracheostomy, and three patients (6.12%) died of complications from an underlying disorder. Two patients (4.1%) in the PEG group had their gastrostomy tube removed owing to successful oral intake.

## 4. Discussion

LG and PEG have various advantages and disadvantages; however, in our study, PEG was shown to be more advantageous than LG when performed simultaneously with LNF.

First, PEG significantly reduced the surgery time than LG. In our study, the median operation time was only 1.81 h in the LNF with PEG group, which was significantly shorter than the operation time of 2.61 h in the LNF with LG group. It is important to note that despite the significantly higher number of patients who needed hiatal hernia repair with LNF in the PEG group (44.9%) vs. the LG group (14.3%), the PEG group still had a significantly shorter operation time. Usually, LG operation times range from 20 min to 1 h, depending on the instrument used, while PEG usually takes 5–10 min.

Second, owing to the short surgical time and minimal incision, the times to start feeding (*p* = 0.005) and reach full feeding (*p* = 0.043) were also shorter in the PEG group; hence, the LOS was shorter in the PEG group (33.5 vs. 16.0 days, *p* = 0.201), although the difference was not significant. Most patients included in this study had one or more severe comorbidities such as neurological and chronic respiratory disorders; therefore, evaluation and analysis of the time to reach full feeding and LOS may have been affected. 

**Figure 2:**
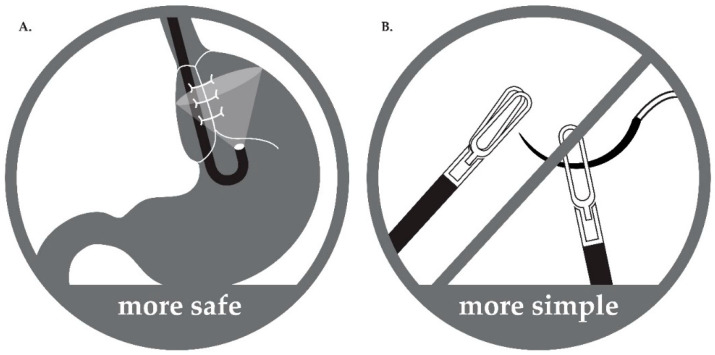
Advantages of the hybrid surgical procedure. (**A**) Safe: During surgery, operator can check tightness and status of fundoplication directly with J turn of the endoscope in stomach. Therefore, complications associated with too tight or too loose wrapping are prevented. (**B**) Simple: As anchoring sutures between stomach and abdominal wall and purse-string sutures are not needed during laparoscopic-assisted percutaneous endoscopic gastrostomy tube insertion, the procedure becomes simpler and easier.

Third, in PEG with LNF, the LNF status can be assessed immediately after surgery. The major complications of LNF are excessive tightening and reinforcement of the lower esophageal sphincter (LES) [8]. PEG performed simultaneously with fundoplication can help in the evaluation of the tightness of the fundoplication at the time of surgery. In our procedure, tightness of the LNF was assessed after LNF and before performing PEG. In cases where the LES that is tightened by fundoplication is too narrow and the endoscope cannot pass, the surgeon can unwind the suture and correct the surgical site upon insertion of the endoscope for PEG, allowing for the evaluation of the degree of fundoplication and bleeding in the stomach after surgery. Therefore, by performing PEG, we can prevent the complications of fundoplication by observing the surgical site and assess whether LNF was successful (Figure 2).

In our institute, surgical prognosis has improved, so more aggressive surgery was performed. The average age and weight at which the surgery was performed wase significantly lower in the PEG group (2.58 years, 10.6 kg) than in the LG group (4.25 years, 14.15 kg). The lowest weight in the PEG group was 4.84 kg, and it has been reported that LNF is also safe for very small children [9]. PEG has also been reported to be safe in children weighing <5 kg [10]. However, when PEG is performed in very small children, tube migration often occurs due to the thinness of the skin and difficulty in selecting a location in the abdomen. Therefore, in this study, patients weighing >5 kg were selected for PEG co-administration. It is expected that as more experience is gained with this procedure, age and weight requirements will be reduced.

Gastrostomy complications have been reported in several studies [4,11,12,13,14,15]. In our study, minor and major complications were infrequent and not significantly different between groups. Overall, minor complications were more common in the PEG group, with a higher incidence of local infection than in the LG group. Furthermore, there were no significant differences in the incidence of major complications. According to a meta-analysis of eight observational studies comparing major complications between children who underwent LG or PEG, major complications were reported to be significantly higher in the PEG group than in the LG group [14]. Mechanical complications were the most common among the major complications that occurred in the PEG group in that analysis; these included accidental tube dislodgement, intraperitoneal tube leakage, and failed tube placement. In our study, the gastrostomy itself was used as a percutaneous endoscopic method, but it was referred to as laparoscopic-assisted PEG since PEG was performed after LNF under laparoscopy. Therefore, failure to mount the tube and complications such as tube dislodgement and intraperitoneal tube leakage did not occur. In addition, the type of tube being mounted is also important to prevent complications such as accidental tube dislodgement or intraperitoneal tube leakage. Currently, the “balloon-type” tube used for LG insertion using the Seldinger technique has considerably reduced operation time, but this technique is not needed in cases of PEG using an “umbrella-type” tube. Moreover, it cannot prevent the balloon from bursting or falling out. These methods may more likely esult in leakage into the abdominal cavity and cause peritonitis, even if the stomach is fixed to the abdominal wall. Although this study did not directly compare the advantages and disadvantages of different catheter types, we recommend using an “umbrella-type” tube when performing gastrostomy for the first time because it can help prevent the balloon from bursting. 

In this study, a 7-month-old female diagnosed with hypoxic ischemic encephalopathy due to birth asphyxia died during hospitalization for surgery. She had recurrent aspiration pneumonia and her clinical course was uneventful at the time of surgery until the fourth postoperative day. She started feeding on the second postoperative day and passed stools on the third postoperative day. She developed a fever on day five post-operation and was diagnosed with *Klebsiella pneumoniae* sepsis and died the following day. In this case, it was not clear whether the operation was a direct cause of death. Nevertheless, it should be noted that more detailed postoperative care is required when actively performing surgery in patients with significant comorbidities.

The disadvantages of this method are the cost of the endoscopy as well as the need for the pediatric endoscopy specialist to perform the operation simultaneously with the pediatric surgeon. When endoscopy is performed immediately after LNF, the direction of the lower esophagus may be different from normal. Therefore, it may be difficult for an inexperienced endoscopist to insert an endoscope through the stomach. In our experience, it is helpful to perform LNF with an inserted nasogastric tube to act as a guide in the entry of the endoscope into the stomach.

This study has several limitations. LGs and PEGs were performed in different patients at different points, so comparison of cross-sectional studies is very limited. Though there is no statistically significant difference, the age, weight, and underlying diseases are not completely identical. Since the follow-up periods of the two groups were different, it was difficult to compare prognosis. Finally, the number of patients was small, and it was a single-center experience. However, even though the PEG group showed low average age and body weight and similar underlying diseases, the PEG group showed shorter operation time and shorter time to start/full feeding. We therefore felt it sufficient to compare the immediate postoperative complications and prognosis.

## 5. Conclusions

Our procedure shows a significantly shorter operation time than conventional surgical gastrostomy. Additionally, endoscopy has the advantage of visually confirming fundoplication. Therefore, we conclude that LNF combined with PEG is a superior method for performing gastrostomy than LG in chronically ill children.

## Figures and Tables

**Table 1:** Baseline characteristics of patients who underwent fundoplication with gastrostomy (*n* = 63).

	Laparoscopic Gastrostomy (*n* = 14)	Percutaneous Endoscopic Gastrostomy (*n* = 49)	*p*-Value
Age, years	4.25 (0.68, 18.26)	2.58 (0.21, 16.4)	0.501
Weight, kg	14.15 (3.90, 25.7)	10.60 (4.84, 46.5)	0.201
Male (%)	9 (64.3)	24 (49.0)	<0.001

Data are presented as the medians (in, max)

**Table 2:** Underlying diseases of patients according to gastrostomy type (*n* = 63).

	Laparoscopic Gastrostomy (*n* = 14) *n* (%)	Percutaneous Endoscopic gastrostomy (*n* = 49) *n* (%)
Neurologic disorders	14 (100)	48 (98.0)
Chronic respiratory disorders	10 (71)	26 (53.1)
Tracheostomy	3 (21.4)	11 (22.4)
Cardiac disorder	1 (7.1)	13 (26.5)
Hiatal hernia (need to repair)	2 (14.3)	22 (44.9)

**Table 3:** Outcomes after surgery according to the gastrostomy type (*n* = 63).

	Laparoscopic Gastrostomy (*n* = 14)	Percutaneous Endoscopic Gastrostomy (*n* = 49)	*p*-Value
Operation time (Median (min, max), hours)	2.61 (1.17, 3.25)	1.81 (1.25, 3.30)	0.001
Time to start feeding (Median (min, max), days)	3.00 (1, 7)	2.00 (1, 18)	0.005
Time to approach full feeding (Median (min, max), days)	9 (2, 52)	6.00 (2, 26)	0.043
Length of stay (Median (min, max), days)	33.50 (6, 121)	16 (6, 578)	0.201

## Data Availability

The data presented in this study are available on request from the corresponding author. The data are not publicly available due to ethical reasons.
